# Green Technique-Solvent Free Microwave Synthesis and Antimicrobial Evaluation of New Thiopyridine Arabinosides

**DOI:** 10.3390/molecules21040477

**Published:** 2016-04-19

**Authors:** Ibrahim A. Maghrabi, Saleh Alghamdi, Majed Alrobaian, Hany A. Eldeab

**Affiliations:** 1 Department of Clinical Pharmacy, College of Pharmacy, Taif University, Taif 5700, Saudi Arabia; t12im@yahoo.com; 2 Department of Microbiology, College of Pharmacy, Taif University, Taif 5700, Saudi Arabia; alamh0@gmail.com (S.A.); majed.alrobaian@hotmail.co.uk (M.A.); 3 Department of Pharmaceutical Chemistry, College of Pharmacy, Taif University, Taif 5700, Saudi Arabia

**Keywords:** *2(*1*H)*-pyridinethione, nucleosides, green synthesis, microwave, solvent-free conditions, antimicrobial

## Abstract

A green protocol has been applied to synthesize a novel series of 3-cyano-2-(tri-*O*-acetyl-β-d-arabinopyranosylthio)pyridines in a short reaction time, in higher yields and with simpler operations, when compared with the conventional heating method. Deacetylation of the obtained acetylated arabinosides produced 2-(β-d-arabinopyranosylthio)-3-cyanopyridines. The structures of the obtained products were confirmed on the basis of spectroscopic data (FT-IR, 1D, 2D-NMR). The synthesized compounds were screened for the antimicrobial activity against a selection of Gram positive and Gram negative bacteria.

## 1. Introduction

In recent years, there has been increasing interest in the synthesis of nucleoside analogues due to their potential use to treat various diseases, such as AIDS, hepatitis, herpes, cancer and microbial infections [1,2,3,4,5,6,7]. On the other hand, thiopyridyl nucleosides have attracted noticeable attention because of their potential function as biological inhibitors, and ligands for carbohydrate-affinity chromatography of enzymes and proteins [8]. Nowadays, the development of green chemical methodologies is one of the most powerful tools in the synthesis of organic substances. Moreover solvent-free microwave irradiation approaches have been utilized to synthesize diverse chemical substances as an efficient green chemistry protocol. It has been reported to be an expeditious, simple, economical and environmentally benign synthetic methodology [9,10,11,12,13,14]. Thus, in continuation of our interest in developing green microwave syntheses of different novel functionalized pyridine compounds and their nucleosides with potentially significant medicinal and pharmacological applications due to their antagonistic activity against human cancer cells and antimicrobial activity [15,16,17], we were prompted to utilize a straightforward microwave-assisted route for the synthesis of thiopyridyl arabinosides as potential antimicrobial agents.

## 2. Result and Discussion

### 2.1. Chemistry

**Scheme 1:**
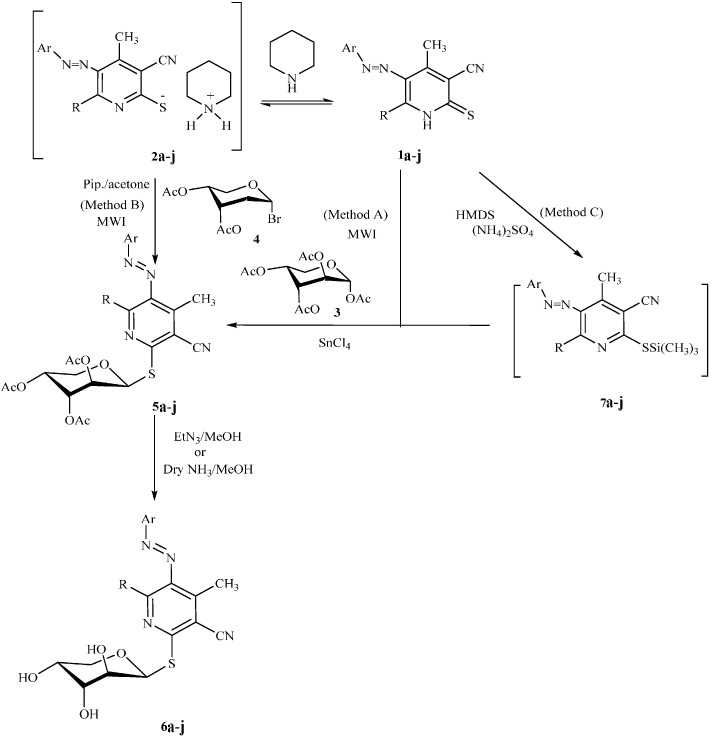
Synthetic pathways of 3-cyano2-(2″,3″,4″-tri-*O*-acetyl-β-d-arabinopyranosylthio) pyridines **5a**–**j**.

In the current report, we targeted dihydropyridine thioarabinosides **5a**–**j** using new, simple and efficient procedures. Three different strategies were used (Scheme 1) and the resulting yields were compared (Table 1). In method A, 3-cyano-2-(2″,3″,4″-tri-*O*-acetyl-β-d-arabinopyranosylthio)-pyridines **5a**–**j** were obtained in high yields by using microwave irradiation (MWI) of a homogenous solid mixture of 2-thiopyridines **1a**–**j** and 1″,2″,3″,4″-tetra-*O*-acetyl-α-d-arabinose (**3**) for 2–3 min using silica gel as a solid support. The solvent-free microwave irradiation method is considered as a green chemistry method offering high chemical yields and milder reaction conditions*.* The reaction of 2-thiopyridines **1a**–**j** with 2″,3″,4″-tri-*O*-acetyl-α-d-arabinopyranosyl bromide (**4**) using a catalytic amount of piperidine in dry acetone/DMF under short duration microwave irradiation also gave the corresponding nucleosides **5a**–**j** in good yield (Method B). On the other hand, the conventional method (method C) involved the reaction of 2-thiopyridines **1a**–**j** and hexamethyldisilazane (HMDS) in the presence of (NH_4_)_2_SO_4_ to give the corresponding 2-trimethylsilylthiopyridines **7a**–**j**. The thiopyridinium salts **7a**–**j** were subsequently treated *in situ* with 1″,2″,3″,4″-tetra-*O*-acetyl-α-d-arabinose (**3**) in the presence of redistilled SnCl_4_ to afford the same target nucleosides **5a**–**j** as the sole nucleoside products. The yields obtained via the microwave irradiation and conventional methods are listed for comparison in Table 1.

The structures of the obtained products **5a**–**j** were confirmed on the basis of their elemental analyses and spectral data (LC-MS/MS, IR, UV, 1D- and 2D-NMR). Thus, the analytical data for **5d** revealed a molecular formula C_26_H_28_N_4_O_7_S (LC-MS (ionization method): *m*/*z* 540 [M]^+^). IR showed signals at υ 1655 and 2230 cm^−1^, assigned to the presence of a carbonitrile group. The ^1^H-NMR spectrum of compound **5d** showed a doublet at δ = 6.36 ppm with the spin-spin coupling constant *J*_H-1″-H-2″_ = 2.8 Hz. This small spin-spin coupling indicates the formation of the β-isomer in ^4^*C*_1_ and ^1^*C*_4_. The use of piperidine as base to abstract hydrogen proton from the 2-thiopyridines **1a**–**j** affords the thiopyridinium salts **2a**–**j** which further attack the anomeric carbon of α-bromoarabinose **4** via equatorial attack. Inversion in the configuration of the anomeric carbon changes the stereochemistry of the obtained products **5a**–**j** to the β-configuration through a S_N_^2^ mechanism. The ^13^C-NMR spectrum showed three signals at δ = 168.9, 170.1 and 170.8 ppm assigned to the acetoxycarbonyl sugar carbon. The 2D-NOESY spectrum showed that H-1″ (δ = 6.36 ppm) had a cross-peak interaction with H-3″ (δ = 5.39 ppm), while no cross peak interaction was observed between the anomeric proton H-1″ and the methyl protons at C-6, indicating that the nucleosidic bond is formed between the anomeric carbon and the oxygen atom at C-2 forming an *S*-nucleoside as sole product. Dry ammonia or triethylamine in methanol were used to convert the protected nucleosides to their corresponding free nucleosides. The yield comparison between the two methods is given in Table 2.

### 2.2. Antimicrobial Activity

**Figure 1:**
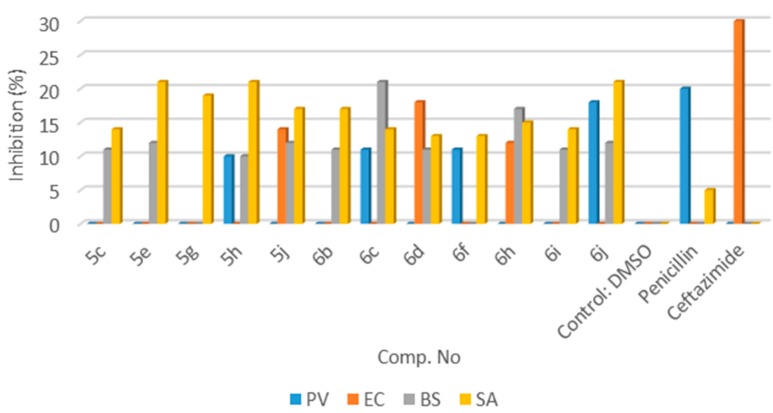
Antimicrobial activity of all synthesized compounds **5a**–**j** and **6a**–**j**.

The antimicrobial activity of the synthesized compounds was investigated against a panel of standard Gram-negative (*Proteus vulgaris *and *Escherichia coli*) and Gram-positive (*Bacillus subtilis *and* Staphylococcus aureus*) bacterial strains (Figure 1). Concerning the antimicrobial activity data in Table 3, some of the synthesized compounds exhibited antibacterial potential comparable to reference drugs such as penicillin and ceftazidime. Concerning the activity against the Gram-positive bacterium *Staphylococcus aureus*, compounds **5j** and **6j** showed higher activity compared to the reference drugs, and compounds **5e**, **6b** and **6h** exhibited good activity, whereas compounds **5c**, **5h**, **6c**, **6e** and **6f** showed moderate activity. Compound **6e** showed good activity against Gram-negative bacterium *Escherichia coli*. On the other hand, compound **6j** showed good activity against *Proteus vulgaris*.

## 3. Materials and Methods

### 3.1. General Information

The microwave synthetic protocol was done using a CEM Microwave system (CEM Corporation, Matthews, VA, USA). Melting points were determined on a (Pyrex capillary) Gallenkamp apparatus (A. Gallenkamp & Co, London, UK). Infrared spectra were recorded with a Nicolet Nexus 470 FT-IR spectrometer (Thermo Scientific, Waltham, MA, USA) in the range 4000–400 cm^−1^ on samples in potassium bromide disks. ^1^H-NMR, ^13^C-NMR and 2D-NMR (COSY, NOESY, ROESY, G-HMBC and G-HMQC) spectra were obtained on a Gemini 400 MHz FT NMR spectrometer (Varian, Agilent Technologies, Edinburgh, UK) in CDCl_3_ and DMSO-*d*_6_; chemical shifts were recorded in δ (ppm) units, relative to Me_4_Si as an internal standard. The mass spectra were recorded on an LCMS-QP 800 LC-MS (Shimadzu, Tokyo, Japan). Elemental analysis data were obtained using a 2400 II series CHN Analyzer (Perkin Elmer, Waltham, MA, USA). Thin-layer chromatography (TLC) was carried out on precoated silica gel F254 plates (Merck, Kenilworth, NJ, USA) and UV light was used for visualization. Column chromatography was performed on a Merck silica gel. The reagents were purchased from Sigma-Aldrich (St. Louis, MO, USA) and used without further purification.

### 3.2 Chemistry

#### 3.2.1. General Procedures for the Synthesis of 3-Cyano-2-(2″,3″,4″-tri-*O*-acetyl-β-d-arabinopyranos-ylthio)-pyridines **5a**–**j**

##### Microwave Method A

A solution of 2-thiopyridine **1a**–**j** (0.01mol) and 1″,2″,3″,4″-tetra-*O*-acetyl-α-d-arabinose (**3**, 0.32 g, 0.01 mol), in a mixture of dichloromethane/methanol (80/20, *v*/*v* %) was treated with silica gel (200–400 mesh, 1.0 g), and then the solvent was removed by evaporation. The solid residue was transferred into a 10-mL vial and irradiated at 200 W power for 2–3 min using the CEM Microwave system (CEM Corporation, Matthews, NC, USA). Purification by flash chromatography (CHCl_3_/cyclohexane, 1:4) was used to afford the desired arabinoside compounds **5a**–**j**.

##### Microwave Method B

To a solution of 2-thiopyridine **1a**–**j** (0.01 mol) and a catalytic amount of piperidine in acetone (5 mL), a solution of 2**″**,3**″**,4**″**-tri-*O*-acetyl-α-d-arabinopyranosyl bromide (**4**, 3.79 g, 0.11 mol) in acetone (5 mL) was added with stirring at room temperature. The mixture was irradiated for a suitable time as shown in Table 1 using the CEM Microwave system and then the solvent was removed under reduced pressure. Flash column was used to purify the product using *n*-hexanes/EtOAc (4:1) as eluent to afford the desired products **5a**–**j**.

##### Conventional Synthesis Method C (Silyl Method)

A mixture of 2-thiopyridine **1a**–**j** (0.01 mol), anhydrous hexamethyldisilazane (HMDS, 25 mL) and a catalytic amount of ammonium sulfate (0.02 g) was stirred and heated under reflux for 48 h. The excess of HMDS was removed under reduced pressure, providing the silylated base as a colorless oil. To a cold solution of the silylated base in dry MeCN (30 mL) a solution of 1″,2″,3″,4″-tetra-*O*-acetyl-α-d-arabinopyranose (**3**, 3.49 g, 11.0 mmol) in dry acetonitrile (10 mL) was added followed by the addition of tin (IV) chloride (1.60 mL, 0.13 mol). The mixture was stirred at 0 °C for 20 min, then at room temperature for an additional 4–8 h until the reaction was completed as judged by TLC analysis, then poured into saturated sodium bicarbonate solution (50 mL) and extracted with chloroform (3 × 50 mL). The extract was dried over anhydrous MgSO_4_, filtered, and concentrated under reduced pressure. Silica-gel chromatography of the residue eluting with gradient MeOH (0%–2%) in CHCl_3_ afforded pure nucleoside **5a**–**j**.

#### 3.2.2. Product Characterization

*3-Cyano-4,6-dimethyl-2-(2″,3″,4″-tri-O-acetyl-β-d-arabinopyranosylthio)-5-phenylazopyridine *(**5a**): MP 132 °C; IR (KBr, cm^−1^) υ 2224 (CN); COSY; ^1^H-NMR (CDCl_3_) δ = 2.04, 2.11 and 2.19 (3 s, 9H, 3CH_3_CO), 2.27 (s, 3H, CH_3_), 2.59 (s, 3H,CH_3_), 3.74, 3.82 (dd, 1H, H-5_a_″, *J* = 3.9, 8.5 Hz), 4.17, 4.28 (dd, 1H, H-5_b_″, *J* = 3.9, 8.5 Hz), 5.33–5.37 (m, 3H, H-2″, H-3″ and H-4″), 6.46 (d, 1H, H-1″, *J_H-1″_*_–*H-2″*_ = 2.3 Hz), 7.34–7.92 (m, 5H, Ar-H); ^13^C-NMR (CDCl_3_) δ = 18.1 (CH_3_), 21.0, 21.1 and 21.2 (3CH_3_CO), 23.4 (CH_3_), 59.7 (C-5″), 65.7 (C-4″), 68.1 (C-3″), 69.0 (C-2″), 92.5 (C-1″), 97.3 (C-3), 113.9 (CN), 122.5–155.1 (Ar-C), 159.1 (C-2), 168.9, 169.6 and 170.4 (3CO); LC-MS (ionization method): *m*/*z* 527 [M + H]^+^; Anal. Calcd. for C_25_H_26_N_4_O_7_S: C, 57.03; H, 4.98; N, 10.64%. Found: C, 57.10; H, 5.01; N, 10.73%.

*3-Cyano-4,6-dimethyl-2-(2″,3″,4″-tri-O-acetyl-β-d-arabinopyranosylthio)-5-(4′-bromophenylazo)pyridine *(**5b**): MP 141 °C; IR (KBr, cm^−1^) υ 2228 (CN); COSY; NOESY; gHMBC; ^1^H-NMR (CDCl_3_) δ = 2.07, 2.15 and 2.25 (3s, 9H, 3CH_3_CO), 2.55 (s, 3H,CH_3_), 2.59 (s, 3H,CH_3_), 3.72, 3.79 (dd, 1H, H-5_a_, *J* = 4.6, 8.5 Hz), 4.10, 4.15 (dd, 1H, H-5_b_, *J* = 4.6, 8.5 Hz), 5.20–5.31 (m, 3H, H-2″, H-3″, H-4″ ); 6.41 (d, 1H, H-1″, *J_H-1″_*_–*H-2″*_ = 2.6 Hz), 7.58 (d, 2H, Ar-H, *J* = 8.7 Hz), 7.76 (d, 2H, Ar-H, *J* = 8.7 Hz); ^13^C-NMR (CDCl_3_) δ = 17.8 (CH_3_), 21.0, 21.5 and 22.0 (3CH_3_CO), 23.3 (CH_3_), 59.5 (C-5″), 65.1 (C-4″), 67.5 (C-3″), 68.8 (C-2″), 92.7 (C-1″), 96.9 (C-3), 113.8 (CN), 123.9–155.2 (Ar-C), 161.1 (C-2), 168.9, 169.8 and 170.2 (3CO); LC-MS (ionization method): *m*/*z* 605 [M + 1]; Anal. Calcd. for C_25_H_25_BrN_4_O_7_S: C, 49.59; H, 4.16; N, 9.25%. Found: C, 49.66; H, 4.05; N, 9.31%.

*3-Cyano-4,6-dimethyl-2-(2″,3″,4″-tri-O-acetyl-β-d-arabinopyranosylthio)-5-(4′-chlorophenylazo)pyridine *(**5c**): MP 129 °C; IR (KBr, cm^−1^) υ 2228 (CN); COSY; NOESY; gHMBC; ^1^H-NMR (CDCl_3_) δ = 2.10, 2.15 and 2.19 (3s, 9H, 3CH_3_CO), 2.55 (s, 3H,CH_3_), 2.59 (s, 3H,CH_3_), 3.74, 3.82 (dd, 1H, H-5_a_, *J* = 3.8, 8.4 Hz), 4.12, 4.18 (dd, 1H, H-5_b_,* J* = 3.8, 8.4 Hz), 5.31–5.39 (m, 3H, H-2″, H-3″ and H-4″), 6.42 (d, 1H, H-1″, *J_H-1″_*_–*H-2″*_ = 2.7 Hz), 7.51(d, 2H, Ar-H, *J* = 4.8 Hz); 7.81 (d, 2H, Ar-H, *J* = 4.8 Hz); ^13^C-NMR (CDCl_3_) δ = 18.2 (CH_3_), 21.1, 21.15 and 22.0 (3CH_3_CO), 23.4 (CH_3_), 60.2 (C-5″), 66.1 (C-4″), 68.1 (C-3″), 68.7 (C-2″), 92.7 (C-1″), 96.8 (C-3), 113.8 (CN), 124.5–155.6 (Ar-C), 160.8 (C-2), 169.6, 170.2 and 170.9 (3CO); LC-MS (ionization method): *m*/*z* 546 [M + 1]; Anal. Calcd. for C_25_H_25_ClN_4_O_7_S: C, 53.52; H, 4.49; N, 9.99%. Found: C, 53.77; H, 4.39; N, 10.06%.

*3-Cyano-4,6-dimethyl-2-(2″,3″,4″-tri-O-acetyl-β-d-arabinopyranosylthio)-5-(4′-methylphenylazo)pyridine *(**5d**): MP 134 °C; IR (KBr, cm^−1^) υ 2230 (CN); COSY; ^1^H-NMR (CDCl_3_) δ = 2.07, 2.15 and 2.19 (3s, 9H, 3CH_3_CO), 2.42 (s, 3H, CH_3_), 2.51 (s, 3H, CH_3_), 2.55 (s, 3H, CH_3_), 3.74, 3.77 (dd, 1H, H-5_a_″, *J* = 2.3, 8.0 Hz), 4.12, 4.17 (dd, 1H, H-5_b_″, *J* = 2.3, 8.0 Hz), 5.36–5.41 (m, 3H, H-2″, H-3″ and H-4″), 6.40 (d, 1H, H-1″, *J_H-1″_*_–*H-2″*_ = 2.7 Hz), 7.31 (d, 2H, Ar-H, *J* = 8.1 Hz); 7.80 (d, 2H, Ar-H, *J* = 8.1 Hz); ^13^C-NMR (CDCl_3_) δ = 17.8 (CH_3_), 21.1, 21.3 and 21.8 (3CH_3_CO), 23.1 (CH_3_), 29.7 (CH_3_), 59.5 (C-5″), 65.7 (C-4″), 67.8 (C-3″), 68.8 (C-2″), 92.4 (C-1″), 96.8 (C-3), 114.1 (CN), 122.3–155.4 (Ar-C), 159.9 (C-2), 168.9, 170.1 and 170.8 (3CO); LC-MS (ionization method): *m*/*z* 540 [M]; Anal. Calcd. for C_26_H_28_N_4_O_7_S: C, 57.77; H, 5.22; N, 10.36%. Found: C, 57.81; H, 5.12; N, 10.20%.

*3-Cyano-4,6-dimethyl-2-(2″,3″,4″-tri-O-acetyl-β-d-arabinopyranosylthio)-5-(4′-methoxyphenylazo)pyridine *(**5e**): MP 142 °C; IR (KBr, cm^−1^) υ 2227 (CN); COSY; ^1^H-NMR (CDCl_3_) δ = 2.05, 2.15 and 2.19 (3s, 9H, 3CH_3_CO), 2.55 (s, 3H, CH_3_), 2.59 (s, 3H, CH_3_), 3.52 (s, 3H, OCH_3_), 3.68, 3.71 (dd, 1H, H-5_a_″, *J* = 2.5, 8.0 Hz), 4.11, 4.16 (dd, 1H, H-5_b_″, *J* = 2.5, 8.0 Hz), 5.35–5.41 (m, 3H, H-2″, H-3″ and H-4″), 6.41 (d, 1H, H-1″, *J_H-1″_*_–*H-2″*_ = 2.7 Hz), 7.31 (d, 2H, Ar-H, *J* = 8.0 Hz); 7.80 (d, 2H, Ar-H, *J* = 8.0 Hz); ^13^C-NMR (CDCl_3_) δ = 21.0, 21.2 and 21.9 (3CH_3_CO), 22.8 (CH_3_), 29.7 (CH_3_), 40.3 (OCH_3_), 59.8 (C-5″), 65.8 (C-4″), 67.8 (C-3″), 69.1 (C-2″), 92.6 (C-1″), 97.3 (C-3), 114.0 (CN), 123.1–155.4 (Ar-C), 160.3 (C-2), 169.5, 170.4 and 170.9 (3CO); LC-MS (ionization method): *m*/*z* 557 [M + 1]; Anal. Calcd. for C_26_H_28_N_4_O_8_S: C, 56.11; H, 5.07; N, 10.07%. Found: C, 56.32; H, 5.19; N, 10.23%.

*3-Cyano-4-methyl-2-(2″,3″,4″-tri-O-acetyl-β-d-arabinopyranosylthio)-5-phenylazo-6-phenylpyridine *(**5f**): MP 177 °C; IR (KBr, cm^−1^) υ 2234 (CN); COSY; ^1^H-NMR (CDCl_3_) δ = 2.14, 2.21 and 2.25 (3s, 9H, 3CH_3_CO), 2.64 (s, 3H, CH_3_), 3.81, 3.86 (dd, 1H, H-5_a_″, *J* = 4.1, 8.5 Hz), 4.19, 4.25 (dd, 1H, H-5_b_″, *J* = 4.1, 8.5 Hz), 5.32–5.38 (m, 3H, H-2″, H-3″ and H-4″), 6.39 (d, 1H, H-1″, *J_H-1″_*_–*H-2″*_ = 2.1 Hz), 7.21–7.76 (m, 10H, Ar-H); ^13^C-NMR (CDCl_3_) δ = 19.1 (CH_3_), 21.3, 21.4 and 21.5 (3CH_3_CO), 60.3 (C-5″), 65.7 (C-4″), 68.3 (C-3″), 69.1 (C-2″), 92.5 (C-1″), 98.3 (C-3), 114.3 (CN), 123.3–147.4 (Ar-C), 152.3 (C-2), 169.2, 169.9 and 170.4 (3CO); LC-MS (ionization method): *m*/*z* 589 [M + 1]; Anal. Calcd. for C_30_H_28_N_4_O_7_S: C, 61.21; H, 4.79; N, 9.52%. Found: C, 61.51; H, 4.83; N, 9.74%.

*3-Cyano-4-methyl-2-(2″,3″,4″-tri-O-acetyl-β-d-arabinopyranosylthio)-6-phenyl-5-(4′-bromophenylazo)-pyridine *(**5g**): MP 183 °C; IR (KBr, cm^−1^) υ 2227 (CN); COSY; ^1^H-NMR (CDCl_3_) δ = 2.15, 2.21 and 2.27 (3s, 9H, 3CH_3_CO), 2.66 (s, 3H, CH_3_), 3.79, 3.85 (dd, 1H, H-5_a_″, *J* = 4.2, 8.1 Hz,), 4.23, 4.29 (dd, 1H, H-5_b_″, *J* = 4.2, 8.1 Hz), 5.35–5.39 (m, 3H, H-2″, H-3″ and H-4″), 6.55 (d, 1H, H-1″, *J_H-1″–H-2″_* = 2.0 Hz), 7.28–7.91 (m, 9H, Ar-H); ^13^C-NMR (CDCl_3_) δ =18.9 (CH_3_), 21.2, 21.3 and 21.4 (3CH_3_CO), 59.8 (C-5"), 65.8 (C-4″), 67.7 (C-3″), 68.9 (C-2″), 93.2 (C-1″), 98.4 (C-3), 114.1 (CN), 124.3–154.9 (Ar-C), 160.0 (C-2), 168.9, 169.7 and 170.8 (3CO); LC-MS (ionization method): *m*/*z* 667 [M + 1]; Anal. Calcd. for C_30_H_27_BrN_4_O_7_S: C, 53.98; H, 4.08; N, 8.39%. Found: C, 54.03; H, 4.19; N, 8.52%.

*3-Cyano-4-methyl-2-(2″,3″,4″-tri-O-acetyl-β-d-arabinopyranosylthio)-6-phenyl-5-(4′-chlorophenylazo)-pyridine *(**5_h_**): MP 172 °C; IR (KBr, cm^−1^) υ 2231 (CN); COSY, NOESY, gHMBC, gHMQC; ^1^H-NMR (CDCl_3_) δ = 2.16, 2.25 and 2.29 (3s, 9H, 3CH_3_CO), 2.65 (s, 3H,CH_3_), 3.80, 3.86 (dd, 1H, H-5_a_″, *J* = 4.0, 8.1 Hz), 4.20, 4.30 (dd, 1 H, H-5_b_″, *J* = 4.0, 8.1 Hz), 5.39–5.42 (m, 3H, H-2″, H-3″ and H-4″), 6.54 (d, 1 H, H-1″, *J_H-1″_*_–*H-2″*_ = 1.9 Hz), 7.25–7.76 (m, 9H, Ar-H); ^13^C-NMR (CDCl_3_) δ = 19.1 (CH_3_), 21.2, 21.3 and 21.4 (3CH_3_CO), 60.1 (C-5″), 65.8 (C-4″), 67.8 (C-3″), 69.0 (C-2″), 93.1 (C-1″), 98.1 (C-3), 113.9 (CN), 123.4–155.1 (Ar-C), 159.8 (C-2), 169.3, 170.2 and 170.6 (3CO); LC-MS (ionization method): *m*/*z* 623 [M + 1]; Anal. Calcd. for C_30_H_27_ClN_4_O_7_S: C, 57.83; H, 4.37; N, 8.99%. Found: C, 57.58; H, 4.61; N, 9.20%.

*3-Cyano-4-methyl-2-(2″,3″,4″-tri-O-acetyl-β-d-arabinopyranosylthio)-6-phenyl-5-(4**′**-methylphenylazo)-pyridine *(**5i**): MP 159 °C; IR (KBr, cm^−1^) υ 2231 (CN); COSY; ^1^H-NMR (CDCl_3_) δ = 2.15, 2.23 and 2.27 (3s, 9H, 3CH_3_CO), 2.47 (s, 3H, CH_3_), 2.63 (s, 3H, CH_3_), 3.78, 3.86 (dd, 1H, H-5_a_″, *J* = 4.1, 8.2 Hz,), 4.22, 4.35 (dd, 1H, H-5_b_″, *J* = 4.1, 8.2 Hz), 5.35–5.39 (m, 3 H, H-2″, H-3″ and H-4″), 6.53 (d, 1 H, H-1″, *J_H-1″_*_–*H-2″*_ = 2.2 Hz), 7.30–7.81 (m, 9H, Ar-H); ^13^C-NMR (CDCl_3_) δ = 18.6 (CH_3_), 21.2, 21.3 and 21.4 (3 CH_3_CO), 22.0 (CH_3_), 60.1 (C-5″), 65.9 (C-4″), 68.2 (C-3″), 68.9 (C-2″), 93.1 (C-1″), 98.1 (C-3), 114.1 (CN), 122.5–154.0 (Ar-C), 159.7 (C-2), 168.5, 169.9 and 170.4 (3CO); LC-MS (ionization method): *m*/*z* 602 [M]; Anal. Calcd. for C_31_H_30_N_4_O_7_S: C, 61.78; H, 5.02; N, 9.30%. Found: C, 61.91; H, 5.20; N, 9.18%.

*3-Cyano-4-methyl-2-(2″,3″,4″-tri-O-acetyl-β-d-arabinopyranosylthio)-6-phenyl-5-(4′-methoxyphenylazo)-pyridine *(**5j**): MP 149 °C; IR (KBr, cm^−1^) υ 2229 (CN); COSY; ^1^H-NMR (CDCl_3_) δ = 2.15, 2.28 and 2.30 (3s, 9H, 3CH_3_CO), 2.61 (s, 3H, CH_3_), 3.65 (s, 3H, OCH_3_), 3.77, 3.87 (dd, 1H, H-5_a_″, *J* = 4.1, 8.1 Hz,), 4.23, 4.35 (dd, 1H, H-5_b_″, *J* = 4.1, 8.1 Hz), 5.37–5.41 (m, 3 H, H-2″, H-3″ and H-4″), 6.51 (d, 1 H, H-1″, *J_H-1″_*_–*H-2″*_ = 2.2 Hz), 7.25–7.80 (m, 9H, Ar-H); ^13^C-NMR (CDCl_3_) δ = 18.7 (CH_3_), 21.2, 21.3 and 21.4 (3CH_3_CO), 45.7 (OCH_3_), 60.2 (C-5″), 65.5 (C-4″), 67.9 (C-3″), 68.9 (C-2″), 93.2 (C-1″), 98.2 (C-3), 114.1 (CN), 122.4–154.2 (Ar-C), 159.9 (C-2), 169.1, 169.8 and 170.4 (3CO); LC-MS (ionization method): *m*/*z* 619 [M + 1]; Anal. Calcd. for C_31_H_30_N_4_O_8_S: C, 60.18; H, 4.89; N, 9.06%. Found: C, 60.25; H, 5.10; N, 9.11%.

#### 3.2.3. General Procedure for Nucleoside Deacetylation: Synthesis of 2-(β-d-Arabinopyranosylthio)-3-cyanopyridines **6a**–**j**

##### Method I

Triethylamine (1.0 mL) was added to a solution of protected arabinoside **5a**–**j** (0.01 mmol) in methanol (10 mL). The mixture was stirred for 12–14 h at room temperature. The solvent was evaporated under reduced pressure and the residue was co-evaporated with methanol until all the triethylamine was removed. The residue was crystallized from an appropriate solvent to give the corresponding deprotected arabinoside **6a**–**j**.

##### Method II

Dry ammonia gas was passed into a solution of protected nucleoside **5a**–**j** (0.5 g) in dry methanol (20 mL) at 0 °C for 30 min. The reaction mixture was stirred until reaction completion as shown by TLC using chloroform/methanol 9/1 as eluent (4–6 h). The resulting mixture was then concentrated under reduced pressure to afford a crude solid. The crude products were purified by silica gel chromatography (chloroform/methanol, 9/1). The products were crystallized from methanol to furnish pure compounds **6a**–**j**.

#### 3.2.4. Product Characterization

*2-(β-d-Arabinopyranosylthio)-3-cyano-4,6-dimethyl-5-phenylazopyridine (***6a**): MP 225 °C; IR (KBr, cm^−1^) υ 3431 (OH), 2237 (CN); COSY; ^1^H-NMR (DMSO-*d*_6_) δ 2.57 (s, 3H, CH_3_), 2.63 (s, 3H, CH_3_), 3.57–3.94 (m, 5H, H-2″, H-3″, H-4″ and 2H-5″), 4.84–5.43 (3OH, exchangeable with D_2_O), 6.04 (d,1H, H-1″, *J_H-1″_*_–*H-2″*_ = 6.5 Hz,), 7.50–7.99 (m, 5H, Ar-H); ^13^C-NMR (DMSO-*d*_6_) δ = 18.8 (CH_3_), 23.2 (CH_3_), 65.9 (C-5″), 67.9 (C-4″), 70.4 (C-3″), 72.8 (C-2″), 96.3 (C-1″), 97.1 (C-3), 114.3 (CN), 121.5–154.8 (Ar-C), 160.8 (C-2); LC-MS (ionization method): *m*/*z* 401 [M + 1]^+^; Anal. Calcd. for C_19_H_20_N_4_O_4_S: C, 56.99; H, 5.03; N, 13.99%. Found: C, 57.17; H, 5.21; N, 14.21%.

*2-(β-d-Arabinopyranosylthio)-3-cyano-4,6-dimethyl-5-(4′-bromophenylazo)pyridine *(**6b**): MP 234 °C; IR (KBr, cm^−1^) υ 3417 (OH), 2228 (CN); COSY; NOESY; gHMBC; ^1^H-NMR (DMSO-*d*_6_) δ = 2.62 (s, 3H, CH_3_), 2.64 (s, 3H, CH_3_), 3.47–3.88 (m, 5H, H-2″, H-3″, H-4″, H-5_a_″ and H-5_b_″), 4.83–5.40 (3OH, exchangeable with D_2_O), 6.03 (d, H-1″, *J_H-1″_*_–*H-2″*_ = 6.9 Hz), 7.69–7.85 (m, 4H, Ar-H); ^13^C-NMR (DMSO-*d*_6_) δ = 18.1 (CH_3_), 23.2 (CH_3_), 65.7 (C-5″), 67.3 (C-4″), 69.4 (C-3″), 72.5 (C-2″), 96.3 (C-1″), 97.6 (C-3), 114.4 (CN), 123.4–155.3 (Ar-C), 161.3 (C-2); LC-MS (ionization method): *m*/*z* 479 [M + 1] Anal. Calcd. for C_19_H_19_BrN_4_O_4_S: C, 47.61; H, 4.00; N, 11.69%. Found: C, 47.76; H, 4.04; N, 11.87%.

*2-(β-d-Arabinopyranosylthio)-3-cyano-4,6-dimethyl-5-(4′-chlorophenylazo)pyridine *(**6c**): MP 241 °C; IR (KBr, cm^−1^) υ 3382 (OH), 2228 (CN); COSY; NOESY; gHMBC; ^1^H-NMR (DMSO-*d*_6_) δ = 2.63 (s, 3H, CH_3_), 2.65 (s, 3H, CH_3_), 3.40–3.89 (m, 5H, H-2″, H-3″, H-4″, H-5a″ and H-5b″), 4.81–5.42 (3OH, exchangeable with D_2_O), 6.10 (d, ,1H, H-1″, *J_H-1″_*_–*H-2″*_ = 6.9 Hz), 7.73 (d, 2H, Ar-H,* J* = 8.9 Hz), 7.93 (d, 2H, Ar-H,* J* = 8.9 Hz); ^13^C-NMR (DMSO-*d*_6_) δ = 18.2 (CH_3_), 23.3 (CH_3_), 65.7 (C-5″), 67.8 (C-4″), 70.1 (C-3″), 72.8 (C-2″), 96.6 (C-1″), 97.5 (C-3), 114.2 (CN), 124.0–155.1 (Ar-C), 161.8 (C-2); LC-MS (ionization method): *m*/*z* 435 [M + 1]^+^; Anal. Calcd. for C_19_H_19_ClN_4_O_4_S: C, 52.47; H, 4.40; N, 12.88%. Found: C, 52.51; H, 4.72; N, 13.03%.

*2-(β-d-Arabinopyranosylthio)-3-cyano-4,6-dimethyl-5-(4′-methylphenylazo)pyridine *(**6d**): MP 210 °C; IR (KBr, cm^−1^) υ 3388 (OH), 2230 (CN); COSY; NOESY; ROESY; gHMBC; gHMQC; ^1^H-NMR (DMSO-*d*_6_) δ = 2.45 (s, 3H, CH_3_), 2.63 (s, 3H, CH_3_), 2.64 (s, 3H, CH_3_), 3.64–3.92 (m, 5H, H-2″, H-3″, H-4″, H-5_a_″ and H-5_b_″), 4.79–5.43 (3OH, exchangeable with D_2_O), 6.03 (d, 1H, H-1″, *J_H-1″_*_–*H-2″*_ = 6.9 Hz), 7.53 (d, 2H, Ar-H,* J* = 8.6 Hz), 7.91 (d, 2H, Ar-H,* J* = 8.6 Hz); ^13^C-NMR (DMSO-*d*_6_) δ = 18.3 (CH_3_), 21.3 (CH_3_), 22.4 (CH_3_), 66.4 (C-5″), 67.7 (C-4″), 70.8 (C-3″), 73.3 (C-2″), 97.0 (C-1″), 98.4 (C-3), 114.2 (CN), 123.3–155.7 (Ar-C), 162.0 (C-2); LC-MS (ionization method): *m*/*z* 414 [M]; Anal. Calcd. for C_20_H_22_N_4_O_4_S: C, 57.96; H, 5.35; N, 13.52%. Found C, 58.20; H, 5.22; N, 13.78%.

*2-(β-d-Arabinopyranosylthio)-3-cyano-4,6-dimethyl-5-(4′-methoxyphenylazo)pyridine* (**6e**): MP 231 °C; IR (KBr, cm^−1^) υ 3381(OH), 2231 (CN); COSY; NOESY; ROESY; gHMBC; gHMQC; ^1^H-NMR (DMSO-*d*_6_) δ = 2.60 (s, 3H, CH_3_), 2.64 (s, 3H, CH_3_), 3.42 (s, 3H, OCH_3_), 3.57–3.93 (m, 5H, H-2″, H-3″, H-4″, H-5_a_″ and H-5_b_″), 4.69–5.40 (3OH, exchangeable with D_2_O), 6.04 (d, 1H, H-1″, *J_H-1″_*_–*H-2″*_ = 6.9 Hz), 7.61 (d, 2H, Ar-H,* J* = 8.6 Hz), 7.97 (d, 2H, Ar-H, *J* = 8.6 Hz); ^13^C-NMR (DMSO-*d*_6_) δ = 21.4 (CH_3_), 21.5 (CH_3_), 46.2 (OCH_3_), 66.5 (C-5″), 67.8 (C-4″), 70.5 (C-3″), 73.2 (C-2″), 96.9 (C-1″), 98.1 (C-3), 114.3 (CN), 123.3–155.7 (Ar-C), 161.6 (C-2); LC-MS (ionization method): *m*/*z* 431 [M + 1]; Anal. Calcd. for C_20_H_22_N_4_O_5_S: C, 55.80; H, 5.15; N, 13.02%. Found C, 55.95; H, 5.81; N, 13.31%.

*2-(β-d-Arabinopyranosylthio)-3-cyano-4-methyl-6-phenyl-5-phenylazopyridine* (**6f**): MP 184 °C; IR (KBr, cm^−1^) υ 3437 (OH), 2227 (CN); COSY; ^1^H-NMR (DMSO-*d*_6_) δ = 2.60 (s, 3H, CH_3_), 3.34–4.32 (m, 5H, H-2″, H-3″, H-4″, H-5_a_″ and H-5_b_″), 4.94–5.40 (3OH, exchangeable with D_2_O), 6.04 (d, 1H, H-1″, *J_H-1″_*_–*H-2″*_= 6.7 Hz), 7.33–7.81 (m, 10 H, Ar-H); ^13^C-NMR (DMSO-*d*_6_) δ = 18.2 (CH_3_), 66.1 (C-5″), 67.4 (C-4″), 70.8 (C-3″), 72.8 (C-2″), 97.8 (C-1″), 97.7 (C-3), 114.3 (CN), 121.8–154.1 (Ar-C), 161.4 (C-2); LC-MS (ionization method): *m*/*z* 463 [M + 1]; Anal. Calcd. for C_24_H_22_N_4_O_4_S: C, 62.32; H, 4.79; N, 12.11%. Found: C, 62.52; H, 4.92; N, 12.40%.

*2-(β-d-Arabinopyranosylthio)-3-cyano-4-methyl-6-phenyl-5-(4′-bromophenylazo)pyridine* (**6g**): MP 172 °C; IR (KBr, cm^−1^) υ 3423 (OH), 2230 (CN); COSY; ^1^H-NMR (DMSO-*d*_6_) δ = 2.56 (s, 3 H, CH_3_), 3.28–3.94 (m, 5H, H-2″, H-3″, H-4″, H-5_a_″ and H-5_b_″), 4.85–5.44 (3OH, exchangeable with D_2_O), 6.05 (d, 1H, H-1″, *J_H-1″_*_–*H-2″*_ = 6.6 Hz), 7.47–7.91 (m, 9H, Ar-H); ^13^C-NMR (DMSO-*d*_6_) δ = 18.4 (CH_3_), 65.8 (C-5″), 67.5 (C-4″), 70.3 (C-3″), 72.7 (C-2″), 97.5 (C-1″), 98.1 (C-3), 114.1 (CN), 123.8–154.2 (Ar-C), 161.4 (C-2); LC-MS (ionization method): *m*/*z* 541 [M + 1]; Anal. Calcd. for C_24_H_21_BrN_4_O_4_S: C, 53.24; H, 3.91; N, 10.35%. Found: C, 53.33; H, 4.01; N, 10.56%. 

*2-(β-d-Arabinopyranosylthio)-3-cyano-4-methyl-6-phenyl-5-(4′-chlorophenyl-azo)pyridine* (**6_h_**): MP 183 °C; IR (KBr, cm^−1^) υ 3422 (OH), 2231 (CN); COSY; ^1^H-NMR (DMSO-*d*_6_) δ = 2.54 (s, 3H, CH_3_), 3.32–3.92 (m, 5H, H-2″, H-3″, H-4″, H-5_a_″ and H-5_b_″), 4.83–5.47 (3OH, exchangeable with D_2_O), 6.05 (d, 1H, H-1″, *J_H-1″_*_–*H-2″*_ = 6.5 Hz), 7.42–7.78 (m, 9H, Ar-H); ^13^C-NMR (DMSO-*d*_6_) δ = 18.7 (CH_3_), 65.5 (C-5″), 67.3 (C-4″), 70.3 (C-3″), 72.7 (C-2″), 97.4 (C-1″), 97.7 (C-3), 114.2 (CN), 122.8–154.3 (Ar-C), 161.1 (C-2); LC-MS (ionization method): *m*/*z* 497 [M + 1]; Anal. Calcd. for C_24_H_21_ClN_4_O_4_S: C, 58.00; H, 4.26; N, 11.27%. Found: C, 58.21; H, 4.43; N, 11.43%.

*2-(β-d-Arabinopyranosylthio)-3-cyano-4-methyl-6-phenyl-5-(4′-methylphenylazo)pyridine (***6i**): MP 182 °C; IR (KBr, cm^−1^) υ 3431 (OH), 2234 (CN); COSY; ^1^H-NMR (DMSO-*d*_6_) δ = 2.41 (s, 3H, CH_3_), 2.53 (s, 3H, CH_3_), 3.38–3.90 (m, 5H, H-2″, H-3″, H-4″, H-5_a_″ and H-5_b_″), 4.47–5.39 (3OH, exchangeable with D_2_O), 6.04 (d, 1H, H-1″, *J_H-1″_*_–*H-2″*_ = 6.4 Hz), 7.31–7.65 (m, 9H, Ar-H); ^13^C-NMR (DMSO-*d*_6_) δ = 18.2 (CH_3_), 21.7 (CH_3_), 65.9 (C-5″), 67.3 (C-4″), 70.4 (C-3″), 71.9 (C-2″), 97.4 (C-1″), 97.9 (C-3), 114.3 (CN), 122.0–153.6 (Ar-C), 161.1 (C-2); LC-MS (ionization method): *m*/*z* 476 [M]; Anal. Calcd. for C_25_H_24_N_4_O_4_S: C, 63.01; H, 5.08; N, 11.76%. Found: C, 62.89; H, 5.22; N, 11.89%.

*2-(β-d-Arabinopyranosylthio)-3-cyano-4-methyl-6-phenyl-5-(4′-methoxyphenylazo)pyridine (***6j**): MP 194 °C; IR (KBr, cm^−1^) υ 3421 (OH), 2229 (CN); COSY; ^1^H-NMR (DMSO-*d*_6_) δ = 2.50 (s, 3H, CH_3_), 3.34–3.84 (m, 5H, H-2″, H-3″, H-4″, H-5_a_″ and H-5_b_″), 4.45 (s, 3H, OCH_3_), 4.79–5.41 (3OH, exchangeable with D_2_O), 6.00 (d, 1H, H-1″, *J_H-1″_*_–*H-2″*_ = 6.6 Hz), 7.21–7.80 (m, 9H, Ar-H); ^13^C-NMR (DMSO-*d*_6_) δ = 18.4 (CH_3_), 45.2 (OCH_3_), 66.1 (C-5″), 67.2 (C-4″), 70.1 (C-3″), 72.4 (C-2″), 97.6 (C-1″), 98.1 (C-3), 113.9 (CN), 122.0–153.6 (Ar-C), 161.2 (C-2); LC-MS (ionization method): *m*/*z* 493 [M + 1]; Anal. Calcd. for C_25_H_24_N_4_O_5_S: C, 60.96; H, 4.91; N, 11.38%. Found: C, 61.07; H, 5.01; N, 11.17%.

### 3.3. Antimicrobial Activity

Controls including the use of the solvent (DMSO) without test compounds showed no antimicrobial activity for this solvent. The antibacterial reference penicillin and ceftazidime discs were tested concurrently as standards. The agar plate disc-diffusion method was used to assess the activity of the compounds. Sterilized filter paper discs (5 mm in diameter) were moistened with the compound solution in dimethyl sulphoxide of specific concentration (10 mg/mL of the compound in DMSO) at 37 °C for 24 h, and the diameter of the clear zone of inhibition surrounding the sample is taken as a measure of the plates were incubated at 37 °C for 48 h with the test discs in place, and the inhibition zones were measured in cm.

## 4. Conclusions

A series novel series of 3-cyano-2-(tri-*O*-acetyl-β-d-arabinopyranosylthio)pyridines **5a**–**j** were obtained utilizing microwave solvent free technique. In contrast to the conventional method, the salient feature of microwave method are rapid reaction rate, high chemical yield, and cleaner reaction condition. Spectroscopic data (FT-IR, 1D, 2D-NMR) revealed a clear structure elucidation for the resulted compounds. Antimicrobial activity of the obtained substances against growth of both G+ and G− tested bacteria has been investigated. 

## Figures and Tables

**Table 1:** Comparison between the microwave and conventional methods in the synthesis of 3-cyano2-(2″,3″,4″-tri-*O*-acetyl-β-d-arabinopyranosylthio) pyridines **5a**–**j**.

Compd. No	R	Ar	Microwave Synthesis		Conventional Synthesis
			Reaction Time/min Yield (%)		Reaction Time/h Yield (%)
			Method A	Method B	Method C
**5a**	CH_3_	C_6_H_5_	3 (86)	7 (82)	56 (44)
**5b**	CH_3_	4-BrC_6_H_4_	3 (88)	7 (84)	55 (42)
**5c**	CH_3_	3-ClC_6_H_4_	3 (85)	6 (81)	55 (45)
**5d**	CH_3_	4-CH_3_C_6_H_4_	2 (90)	7 (85)	56 (47)
**5e**	CH_3_	4-OCH_3_C_6_H_4_	2 (90)	6 (85)	56 (51)
**5f**	C_6_H_5_	C_6_H_5_	2 (88)	6 (87)	56 (41)
**5g**	C_6_H_5_	4-BrC_6_H_4_	2 (95)	7 (90)	55 (52)
**5h**	C_6_H_5_	4-ClC_6_H_4_	2 (93)	6 (88)	55 (55)
**5i**	C_6_H_5_	4-CH_3_C_6_H_4_	2 (89)	8 (81)	57 (49)
**5j**	C_6_H_5_	4-OCH_3_C_6_H_4_	2 (95)	8 (90)	56 (53)

**Table 2:** Yield comparison between triethylamine (Method I) and dry ammonia (Method II).

Compd. No.	R	Ar	Method I Yield %	Method II Yield %
**6a**	CH_3_	C_6_H_5_	90	78
**6b**	CH_3_	4-BrC_6_H_4_	92	81
**6c**	CH_3_	3-ClC_6_H_4_	90	83
**6d**	CH_3_	4-CH_3_C_6_H_4_	91	80
**6e**	CH_3_	4-OCH_3_C_6_H_4_	89	81
**6f**	C_6_H_5_	C_6_H_5_	86	77
**6g**	C_6_H_5_	4-BrC_6_H_4_	81	79
**6h**	C_6_H_5_	4-ClC_6_H_4_	84	76
**6i**	C_6_H_5_	4-CH_3_C_6_H_4_	90	81
**6j**	C_6_H_5_	4-OCH_3_C_6_H_4_	88	80

**Table 3:** Antimicrobial activity of all synthesized compounds **5a**–**j** and **6a**–**j**.

Compd. No	R^1^	R^2^	Ar	Inhibition (%)			
				*PV*	*EC*	*BS*	*SA*
**5a**	CH_3_	CH_3_	C_6_H_5_	0.0	0.0	0.0	0.0
**5b**	CH_3_	CH_3_	4-BrC_6_H_4_	0.0	0.0	0.0	0.0
**5c**	CH_3_	CH_3_	4-ClC_6_H_4_	0.0	0.0	11	14
**5d**	CH_3_	CH_3_	4-CH_3_C_6_H_4_	0.0	0.0	0.0	0.0
**5e**	CH_3_	CH_3_	4-OCH_3_C_6_H_4_	0.0	0.0	12	15
**5f**	C_6_H_5_	CH_3_	C_6_H_5_	0.0	0.0	0.0	0.0
**5g**	C_6_H_5_	CH_3_	4-BrC_6_H_4_	0.0	0.0	0.0	13
**5h**	C_6_H_5_	CH_3_	4-ClC_6_H_4_	10	0.0	10	14
**5i**	C_6_H_5_	CH_3_	4-CH_3_C_6_H_4_	0.0	0.0	0.0	0.0
**5j**	C_6_H_5_	CH_3_	4-OCH_3_C_6_H_4_	0.0	14	12	17
**6a**	CH_3_	CH_3_	C_6_H_5_	0.0	0.0	0.0	0.0
**6b**	CH_3_	CH_3_	4-BrC_6_H_4_	0.0	0.0	11	15
**6c**	CH_3_	CH_3_	4-ClC_6_H_4_	11	0.0	12	14
**6d**	CH_3_	CH_3_	4-CH_3_C_6_H_4_	0.0	0.0	0.0	0.0
**6e**	CH_3_	CH_3_	4-OCH_3_C_6_H_4_	0.0	18	11	13
**6f**	C_6_H_5_	CH_3_	C_6_H_5_	11	0.0	0.0	13
**6g**	C_6_H_5_	CH_3_	4-BrC_6_H_4_	0.0	0.0	0.0	0.0
**6h**	C_6_H_5_	CH_3_	4-ClC_6_H_4_	0.0	12	17	15
**6i**	C_6_H_5_	CH_3_	4-CH_3_C_6_H_4_	0.0	0.0	11	12
**6j**	C_6_H_5_	CH_3_	4-OCH_3_C_6_H_4_	18	0.0	12	21
Control: DMSO				0.0	0.0	0.0	0.0
Penicillin				20	0.0	0.0	14
Ceftazidime				0.0	30	0.0	0.0
